# Supplementary material for: Schnurri-3 inhibition rescues skeletal fragility and vascular skeletal stem cell niche pathology in the OIM model of osteogenesis imperfecta
Source: Bone Res. 2024 Aug 26;12:46. doi: 10.1038/s41413-024-00349-1 (PMC11345453; doi:10.1038/s41413-024-00349-1)
Supplement: Supplementary file 1 — supplementary data [file 41413_2024_349_MOESM1_ESM.docx]

**
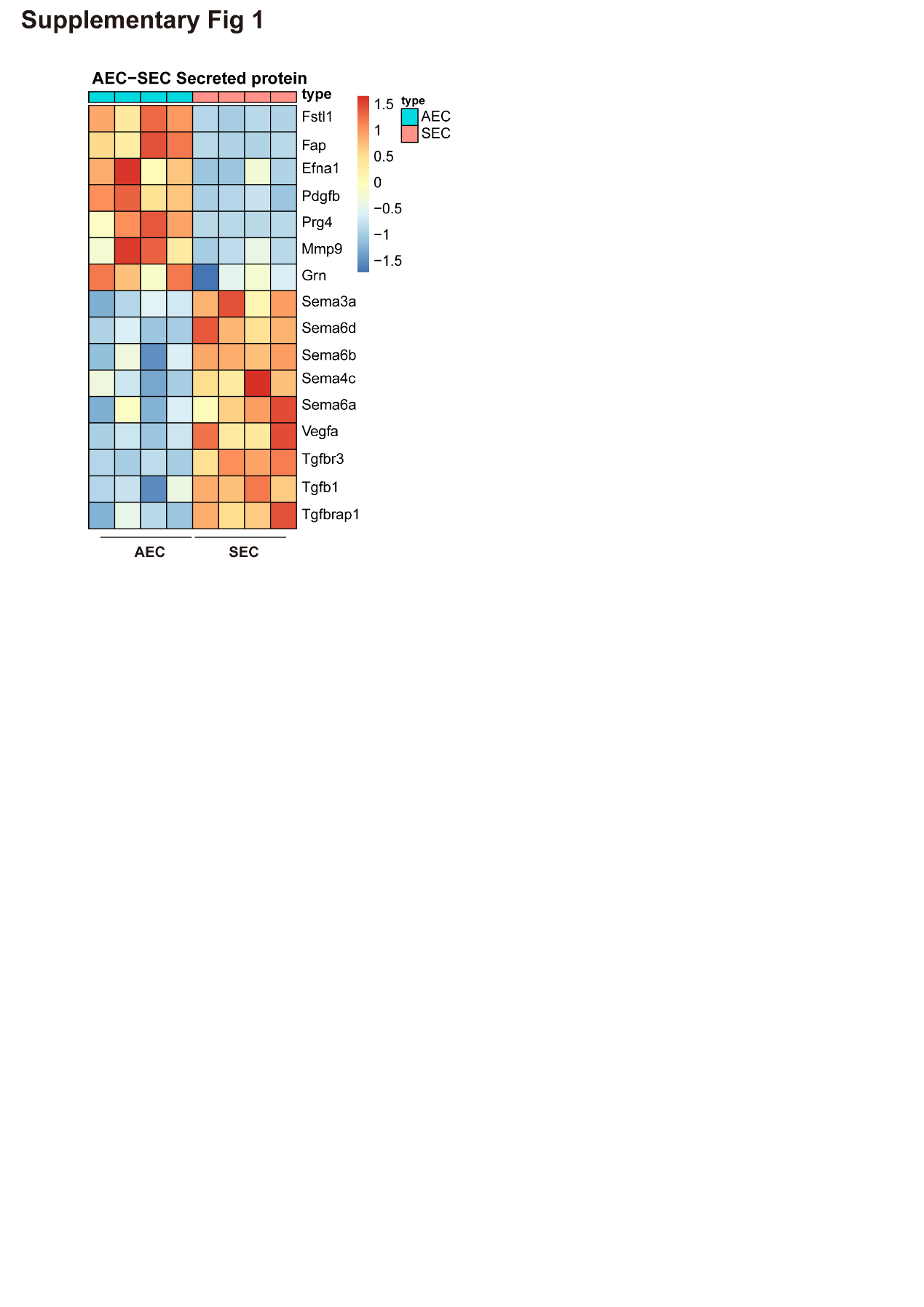
**

**Supplementary Figure 1.**

Heatmap of differential expression of Secreted Genes Between AEC and SEC in 2months Mice. AEC: Arterial Endothelial Cells; SEC: Sinusoidal Endothelial Cells.

**
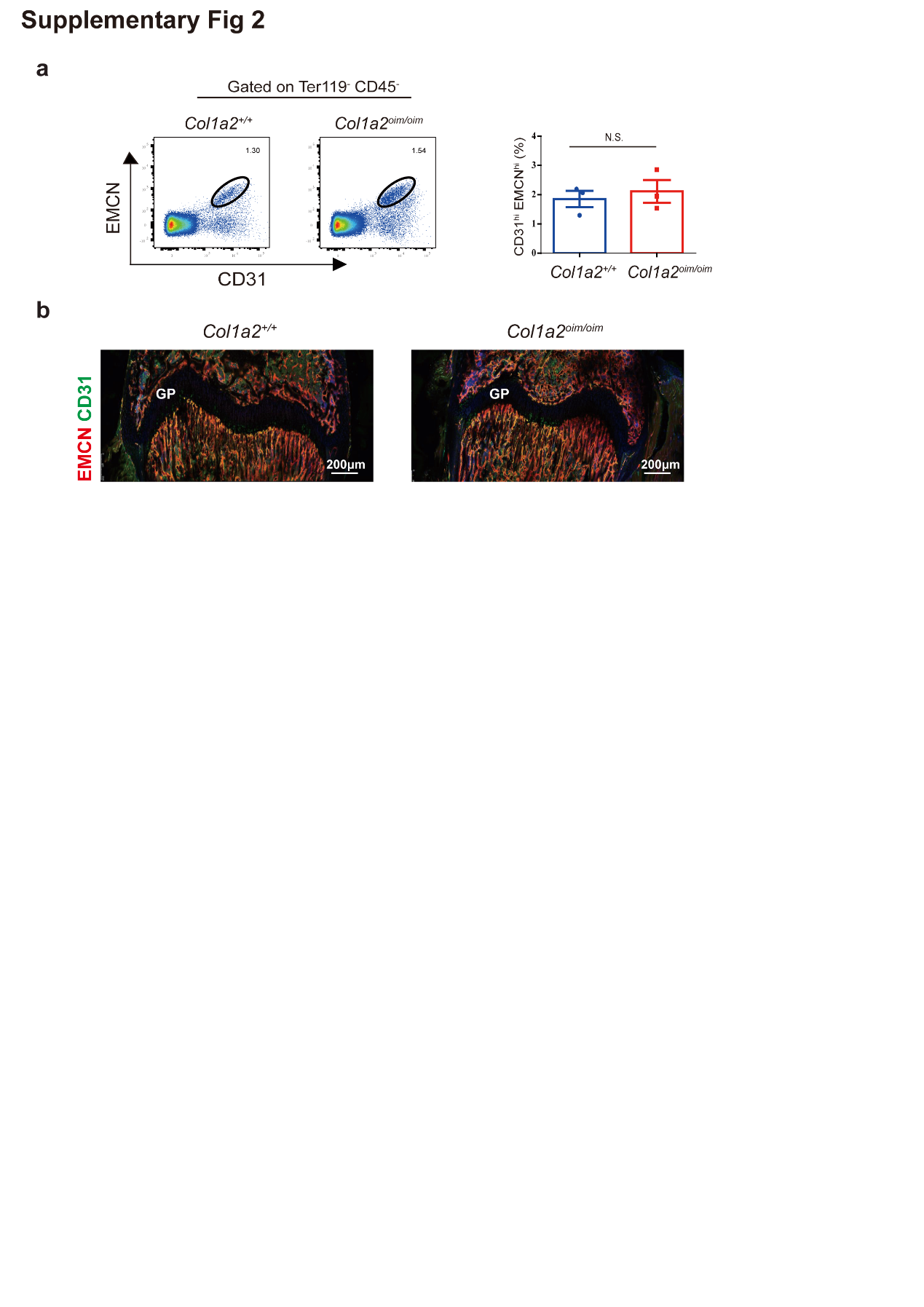
**

**Supplementary Figure 2.** ***Col1a2^oim/oim^* mice have normal levels of CD31^hi^ EMCN^hi^ endothelium**

(a) Representative flow cytometry plots and relative frequency of CD31^hi^EMCN^hi^ endothelial cells from the femurs of 3-week-old male *Col1a2^+/+^* and *Col1a2 ^oim/oim^* mice (*n =* 3)

(b) Representative confocal images (*n* = 3 total images per group) of EMCN (Green) and CD31 (Red) immunostained sections from the femurs of 3-week-old *Col1a2^+/+^*and *Col1a2 ^oim/oim^* male mice. The growth plate is labeled. Scale bars, 100 μm


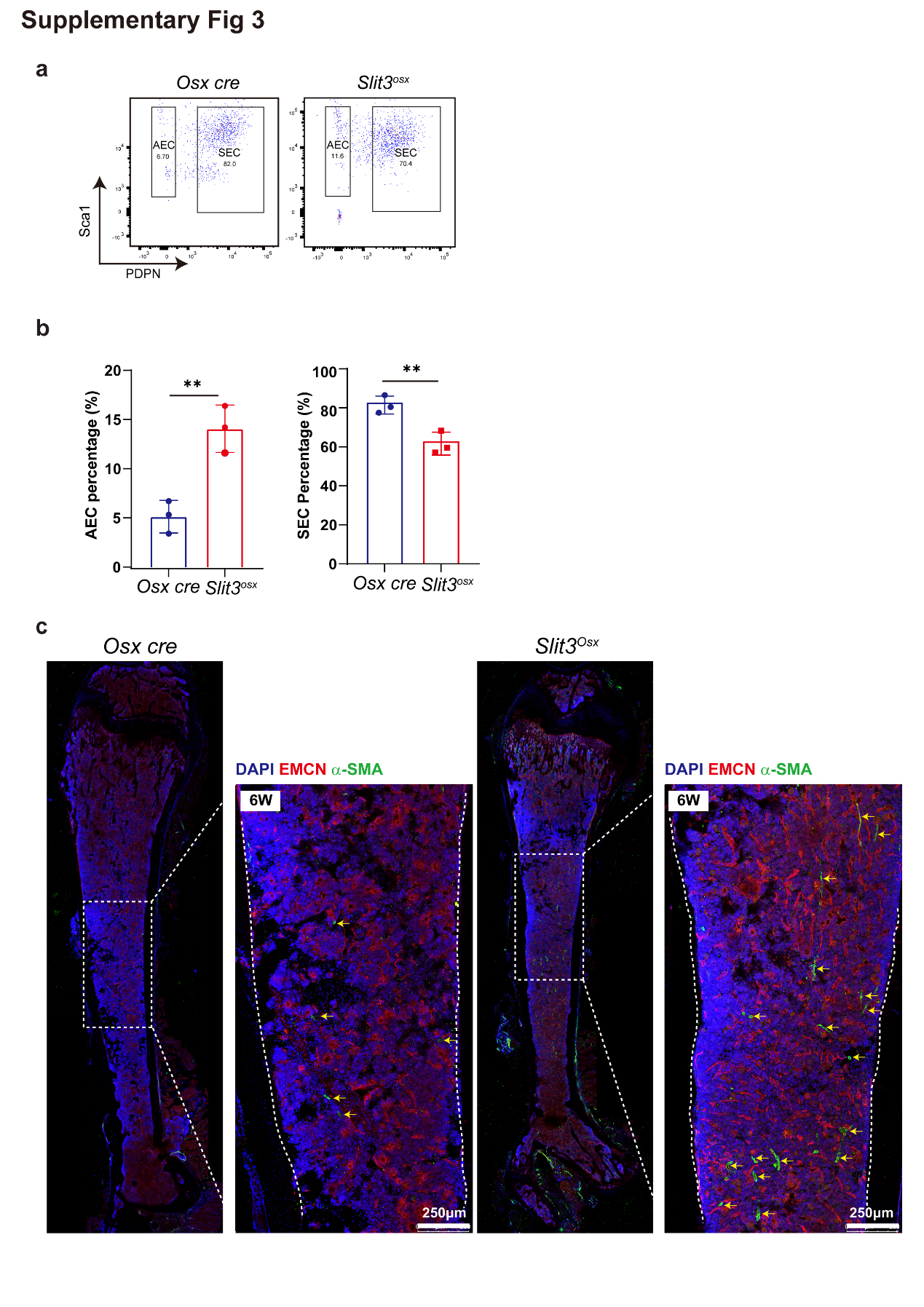


**Supplementary Figure 3.** ***Slit3^osx^* mice have pathological extension of AECs**

(a) Representative flow cytometry plots and (b) quantitative analysis of arterial endothelial cells (AECs) and sinusoidal endothelial cells (SECs).

(c) Representative confocal images of femur sections from 6-week-old *Osx cre* and *Slit3^osx^* male mice stained with EMCN (Red) and α-SMA (Green). Scale bars, 250μm. Results are presented as mean ± s.e.m.; **P < 0.01 by an unpaired two-tailed Student’s t-test in all panels.

**
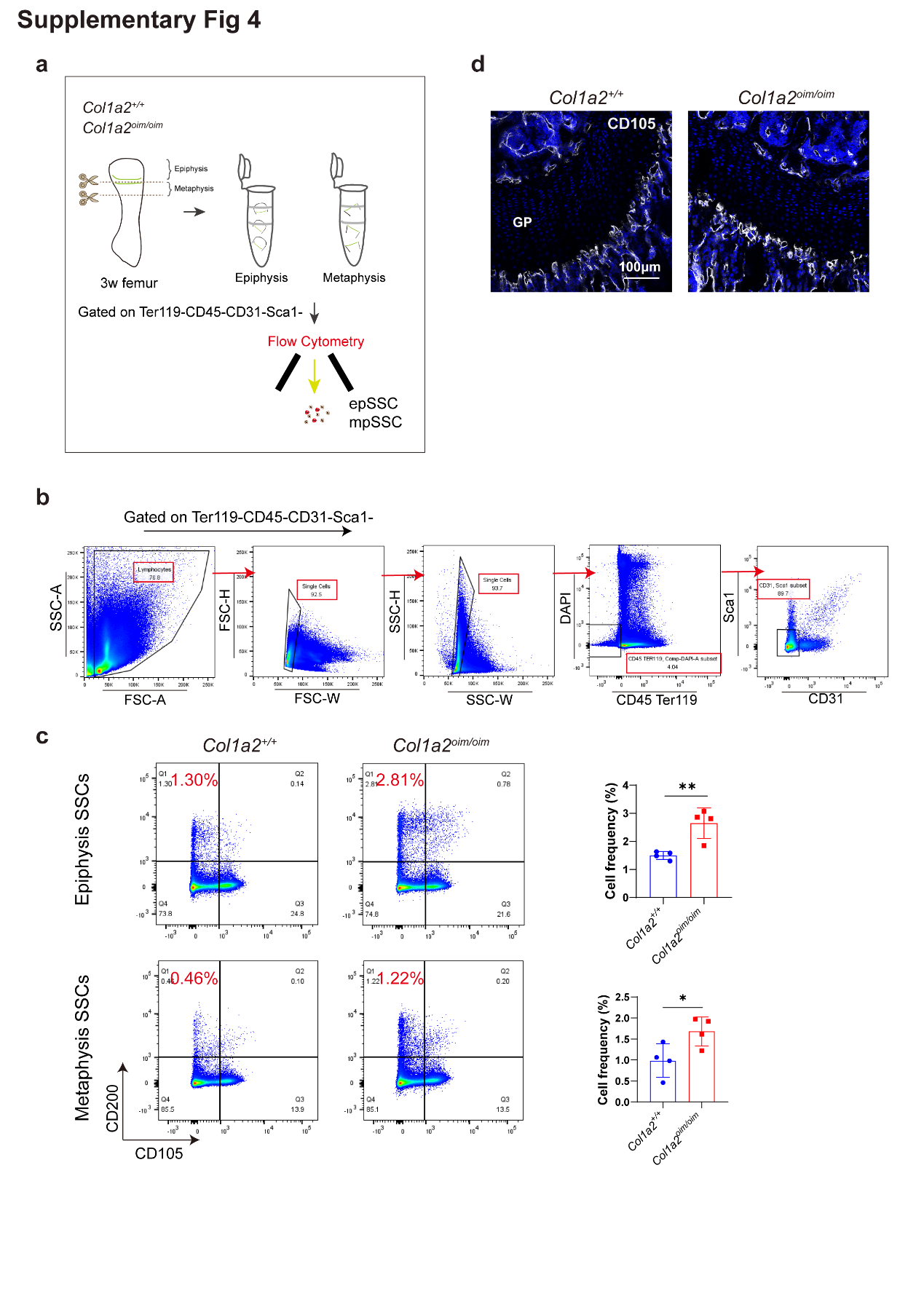
**

**Supplementary Figure 4.**

1. Schematic diagram depicting the strategy for flow cytometry of SSCs.

(b) Gating strategy for flow cytometry of SSCs in femur with *Col1a2^+/+^* and *Col1a2 ^oim/oim^* mice.

(c) Representative flow cytometry plots of SSCs from 3w *Col1a2^+/+^* and *Col1a2 ^oim/oim^* mice in different anatomical locations, Plots are representative of 4 independent experiments.

(d) Representative confocal images (*n* = 3 total images per group) of CD105 (White) immunostained sections from the femurs of 3-week-old *Col1a2^+/+^*and *Col1a2 ^oim/oim^* male mice. Scale bars, 100 μm


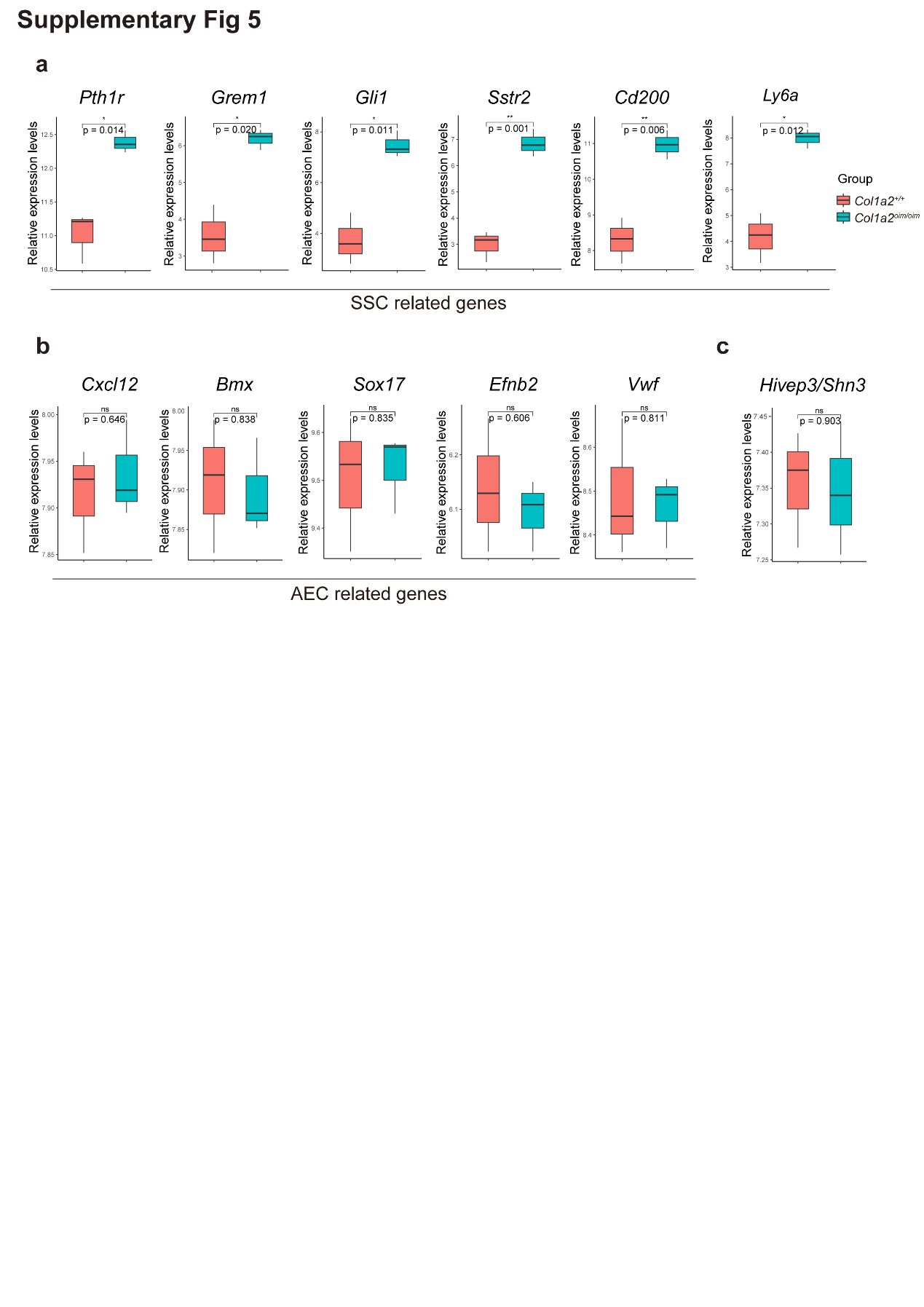


**Supplementary Figure 5. Bulk RNA-seq analysis of *Col1a2^+/+^* and *Col1a2^oim/oim^***

(a)The box diagram is shown that SSC related genes expression in *Col1a2^+/+^* and *Col1a2^oim/oim^* mice

(b) The box diagram is shown that AEC related genes expression in *Col1a2^+/+^* and *Col1a2^oim/oim^* mice

(c) The box diagram is shown that *Hivep3/Shn3* expression in *Col1a2^+/+^* and *Col1a2^oim/oim^* mice

**
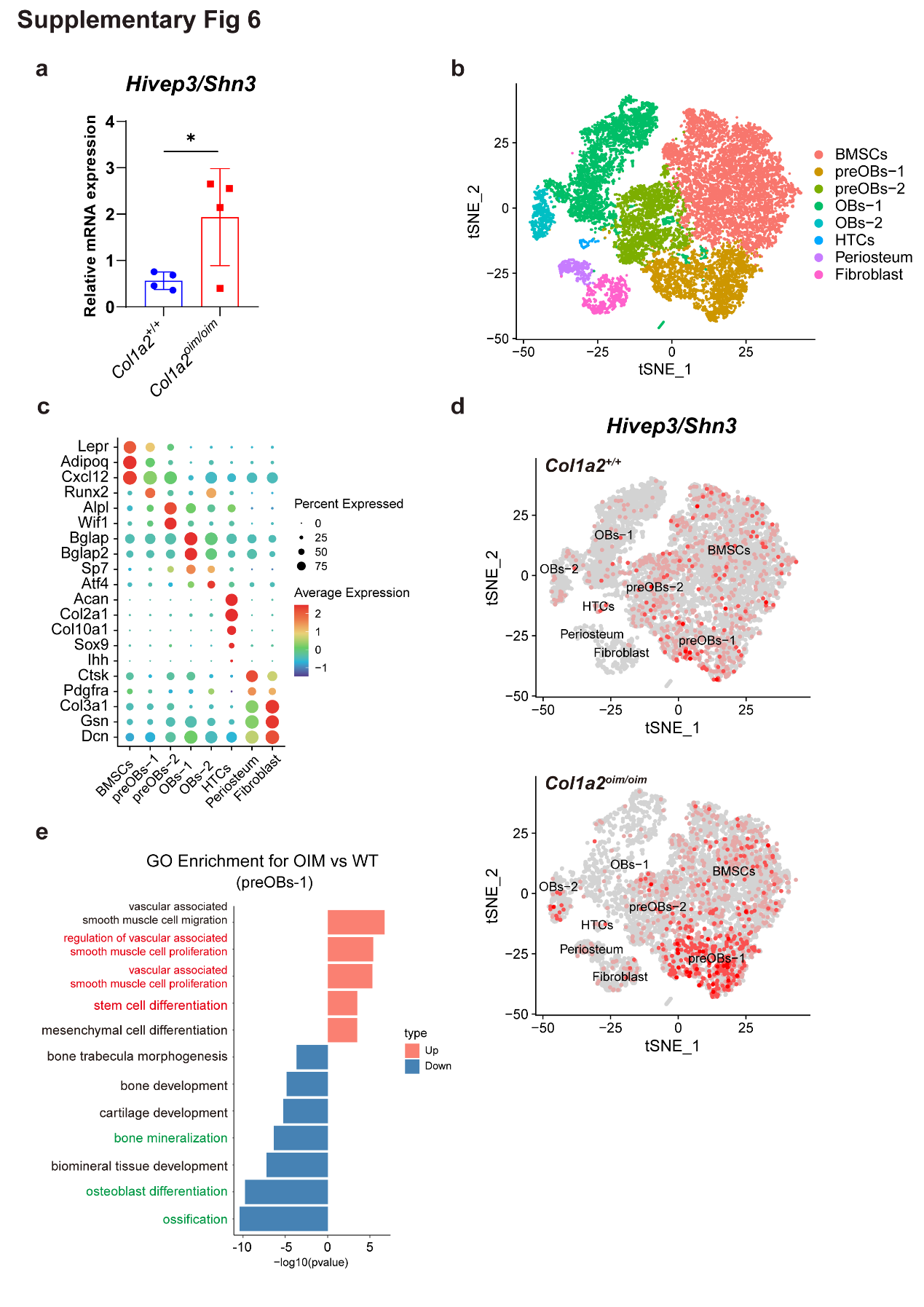
**

**Supplementary Figure 6. Single-cell transcriptomic analysis of *Col1a2^+/+^ and Col1a2^oim/oim^***

(a) The transcription expression levels of *Hivep3/Shn3* were assessed in femur bone RNA and normalized to *hprt* from 3-week-old *Col1a2^+/+^* and *Col1a2 ^oim/oim^* male mice (n =4 mice per group).

(b) The t-distributed stochastic neighbor embedding (t-SNE) plot of the 8 identified main cell types in *Col1a2^+/+^ and Col1a2^oim/oim^* mice.

(c) Dot plots showing the 20 signature gene expressions across the 8 cellular clusters. The size of dots represents the proportion of cells expressing the particular marker, and the spectrum of color indicates the mean expression levels of the markers.

(d) tSNE plots showing the gene of *Hivep3/Shn3* from *Col1a2^+/+^ and Col1a2^oim/oim^*

(e) Bar plot showing the enriched Gene Ontology enrichment of differentially expressed genes between *Col1a2^+/+^ and Col1a2^oim/oim^* from preOBs-1 cellular clusters .

**
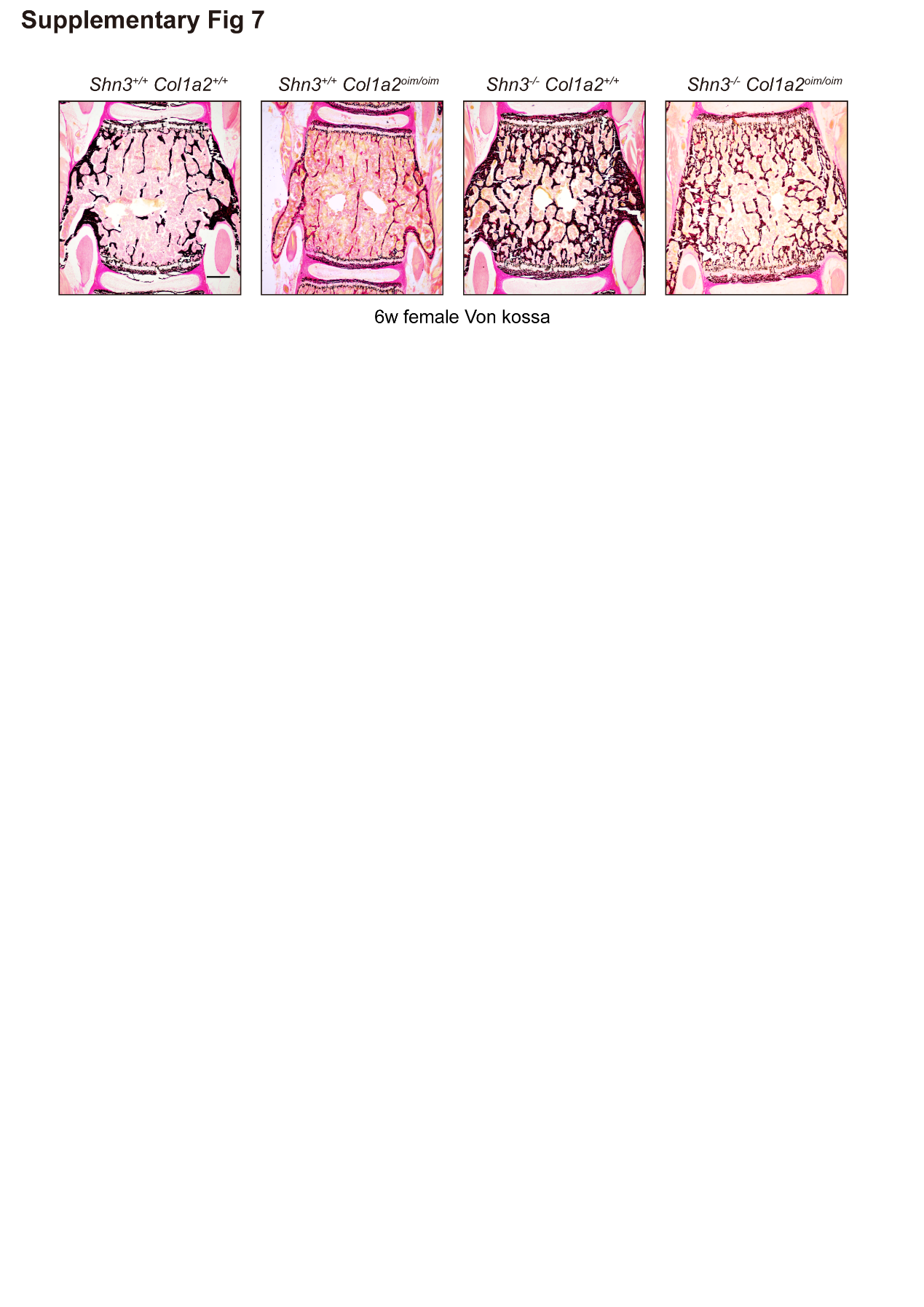
**

**Supplementary Figure 7. Deletion of SHN3 improves bone mass in female *Col1a2^oim/oim^* mice**

Representative images of Von Kossa staining of L3 vertebrae in *Shn3*^+/+^*Col1a2^+/+^*and *Shn3*^+/+^*Col1a2^oim/oim^* and *Shn3*^-/-^*Col1a2^+/+^* and *Shn3*^-/-^*Col1a2 ^oim/oim^* female mice at 6 weeks of age, Scale bars, 500 μm.


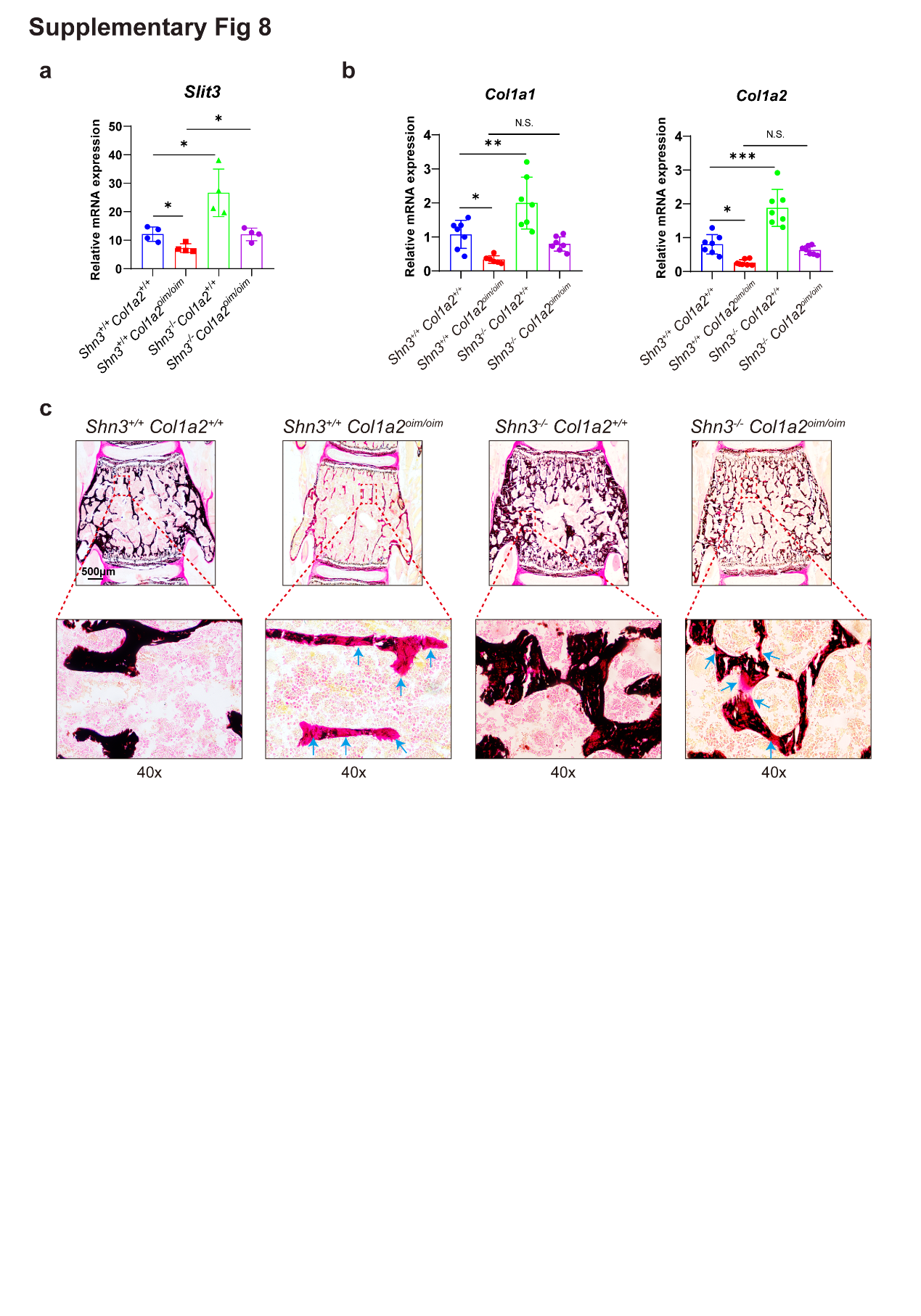


**Supplementary Figure 8.** **SHN3 deficiency does not altered the pathological collagen structure in the bone of *Col1a2^oim/oim^* mice**

(a) The transcription expression levels of *Slit3* were assessed in femur bone RNA and normalized to *hprt* from 3-week-old *Shn3^+/+^Col1a2^+/+^* and *Shn3^+/+^Col1a2 ^oim/oim^* and *Shn3^-/-^Col1a2^+/+^* and *Shn3^-/-^Col1a2 ^oim/oim^* male mice (n =4 mice per group).

(b) The transcription expression levels of *Col1a1* and *Col1a2* were assessed in femur bone RNA and normalized to *hprt* from 3-week-old *Shn3^+/+^Col1a2^+/+^* and *Shn3^+/+^Col1a2 ^oim/oim^* and *Shn3^-/-^Col1a2^+/+^* and *Shn3^-/-^Col1a2 ^oim/oim^* male mice (n =7 mice per group).

(c)Representative images of Von Kossa staining of L3 vertebrae in *Shn3*^+/+^*Col1a2^+/+^*and *Shn3*^+/+^*Col1a2^oim/oim^* and *Shn3*^-/-^*Col1a2^+/+^* and *Shn3*^-/-^*Col1a2 ^oim/oim^* female mice at 6 weeks of age. Scale bars, 500 μm. Below shows 40x magnification of trabecular bone to represent the image. Arrows indicate incomplete ossification osteoid.


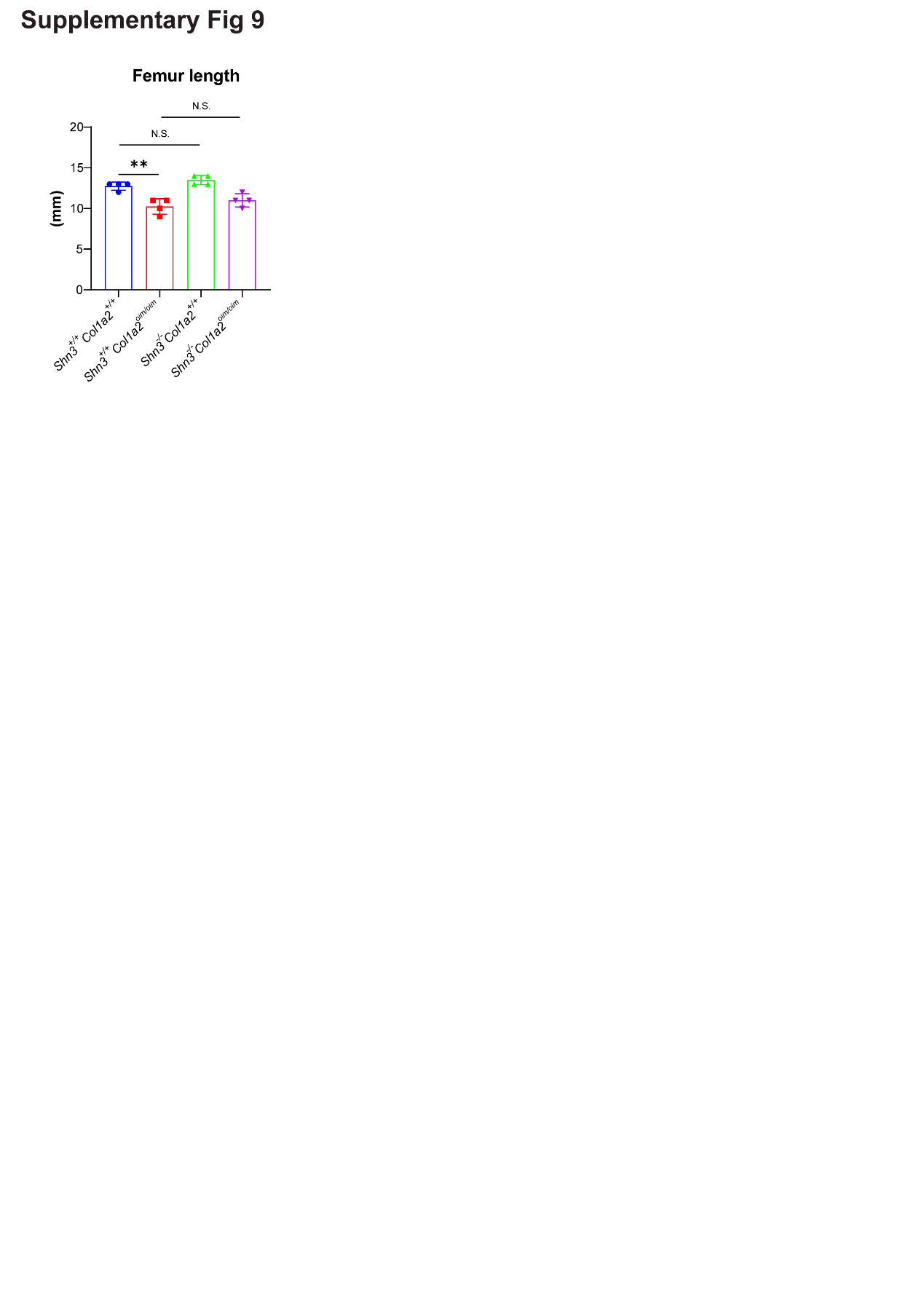


**Supplementary Figure 9.** **Deletion of SHN3 is dispensable for the bone length on *Col1a2^oim/oim^* mice**

Relative quantification of bone length in *Shn3^+/+^Col1a2^+/+^* and *Shn3^+/+^Col1a2 ^oim/oim^* and *Shn3^-/-^Col1a2^+/+^* and *Shn3^-/-^Col1a2 ^oim/oim^* male mice at 6 weeks of age.


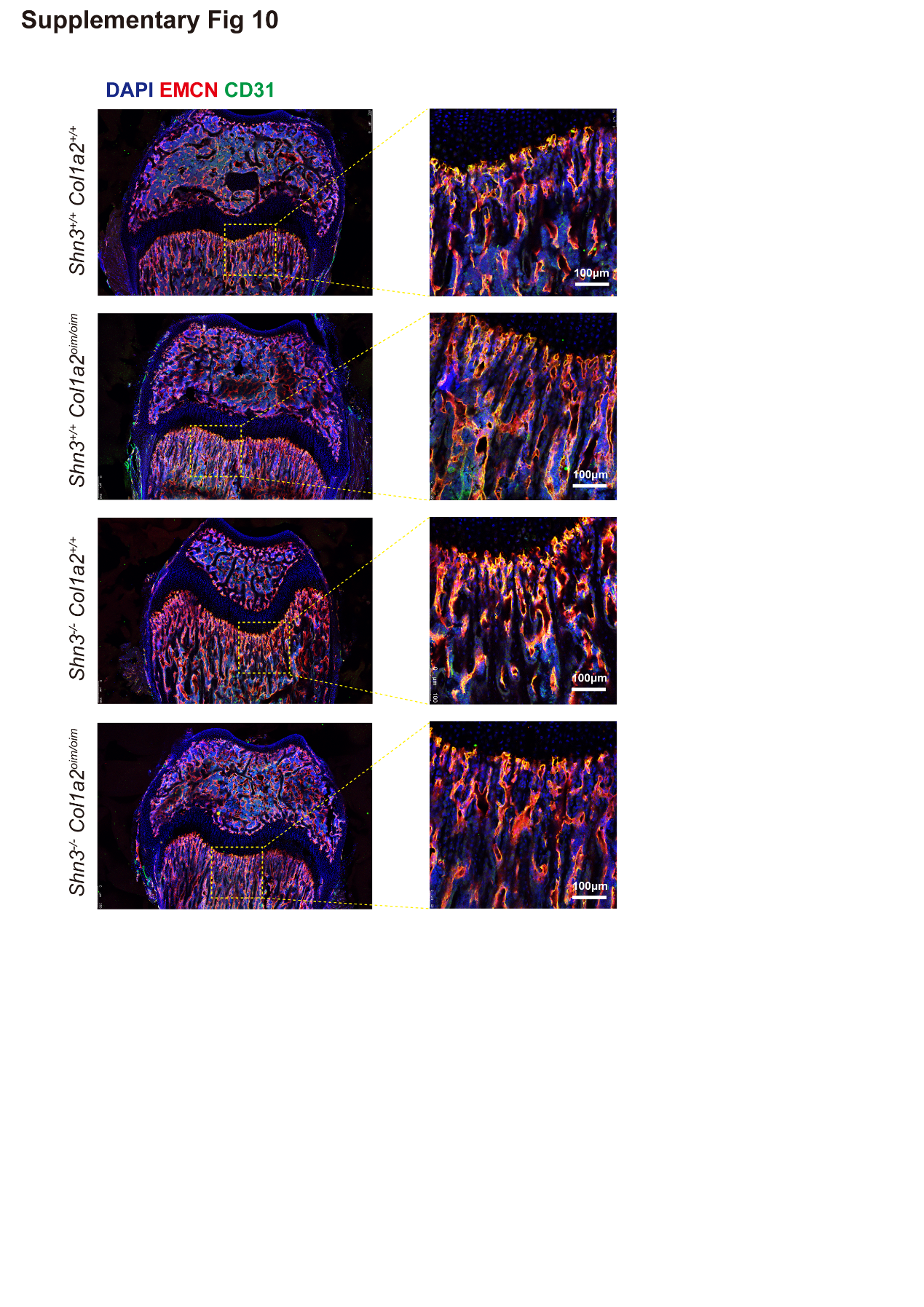


**Supplementary Figure 10. Comparison of Type-H blood vessels in four groups of mice**

Representative confocal images of femur sections from 3-week-old *Shn3^+/+^Col1a2^+/+^* and *Shn3^+/+^Col1a2 ^oim/oim^* and *Shn3^-/-^**Col1a2^+/+^* and *Shn3^-/-^Col1a2 ^oim/oim^* mice stained with EMCN (Red) and CD31 (Green). Scale bars, 100μm. Right shows magnification of Type-H blood vessels to represent the image.


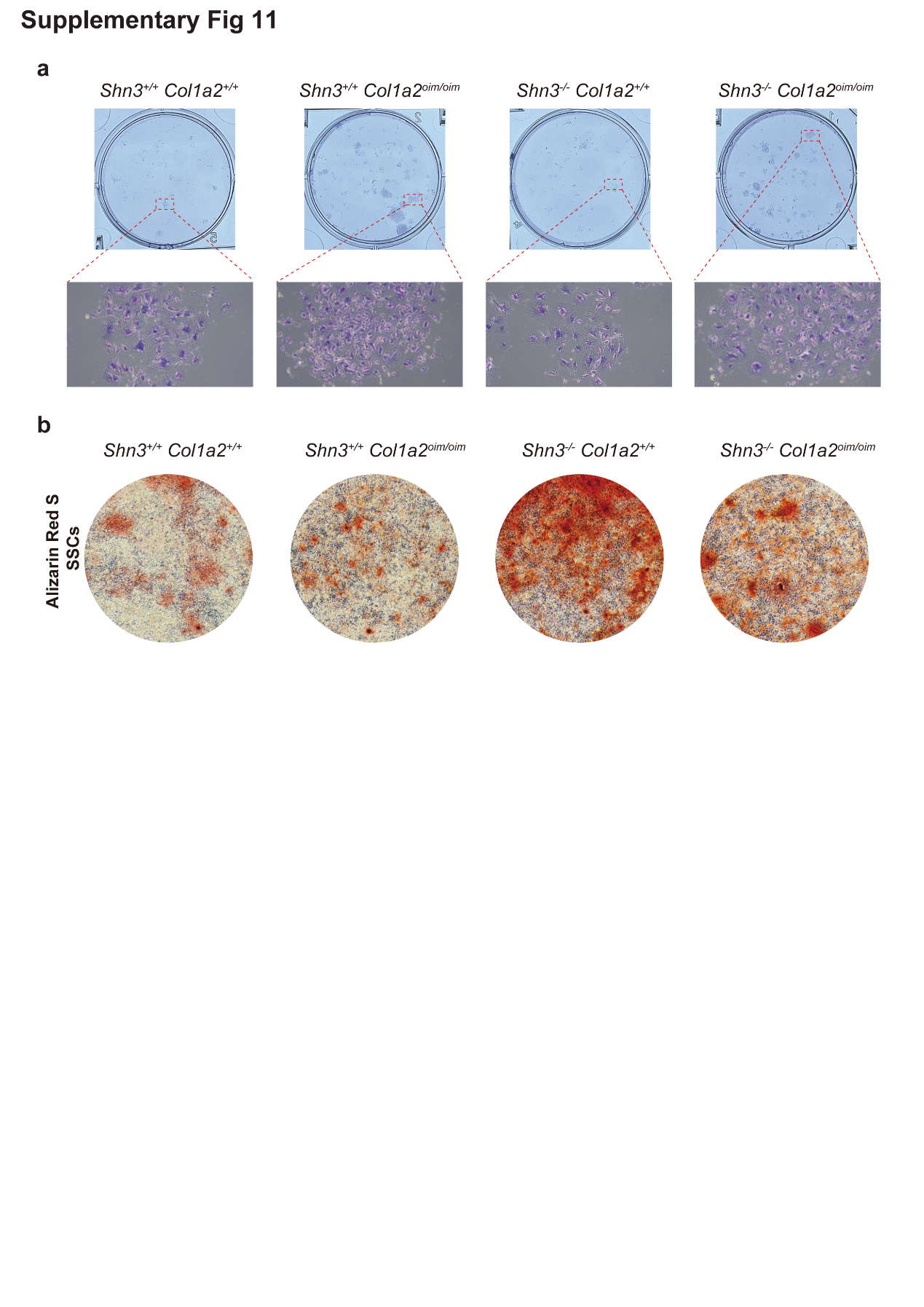


**Supplementary Figure 11.** **Deletion of SHN3 no change high proliferation of SSCs though enhanced the osteoblast differentiation in *Col1a2^oim/oim^* mice**

(a) Representativeimages of colony formation of SSCs sorted from *Shn3^+/+^Col1a2^+/+^* and *Shn3^+/+^Col1a2 ^oim/oim^* and *Shn3^-/-^Col1a2^+/+^* and *Shn3^-/-^Col1a2 ^oim/oim^* mice (upper) and colony forming magnified images (below) are shown.

(b) Representative images of Alizarin Red S Staining (ARS) of SSCs sorted from *Shn3^+/+^Col1a2^+/+^* and *Shn3^+/+^Col1a2 ^oim/oim^* and *Shn3^-/-^Col1a2^+/+^* and *Shn3^-/-^Col1a2 ^oim/oim^* mice are shown.
